# Aids to Poor Sufferers

**Published:** 1906-06-16

**Authors:** 


### AIDS TO POOR SUFFERERS.

### SOME INVALID CONTRIVANCES.

The weariness of long illness can be lessened by contrivances for moving the patient, but many of the machines are so expensive as to be out of the reach of sufferers of the poorer classes. Therefore it is worth while to tell of some cheap and simple things, such as can be made at home by anyone with a little ingenuity. They are all things that have been used by invalids, and by them described to another sufferer. One lady whose spine is injured, and who is bedridden for life, is able to be transferred from her bed to a couch with little trouble to her nurse, or pain to herself, by means of a network of strong tape woven in and out, and fastened at every crossing. At each side are loops through which stout bars of wood can be passed. This network lies on the bed under the sheet. When the invalid is to be moved the wooden rods are passed through the loops, and two members of the family can, by taking hold of these, lift the patient from the bed to a couch which is placed at the foot of the bed—from which the high metal end has been removed and replaced by a low footboard. While the patient is on the couch another netting is placed upon the bed, so that when the next move is made there is no painful pushing of the stretcher under. This plan could be utilised also for transferring a patient from one bed to another, and in this connection we may say that where the accommodation permits it is often less fatiguing to put the patient in another bed than to make the bed with the patient in it, and the change to the other bed, which has been aired for a day, is in itself refreshing.

Another invalid, a poor woman, had her lot lightened by the possession of a bed-table, which her husband made for a few shillings. This table is reversible, and is so arranged by means of the supports on each side that one side slopes, and can be used for reading and writing, while the other is flat, for use when eating or sewing; for this invalid helps to maintain herself by sewing. She wrote to a sympathiser: "I have one of those little sewing-machines that screw to the table and are run by a crank, and I manage to make two dozen pairs of overalls a week. I have made twice that number some weeks. . . . So I can contribute something to the family expenses. And the work makes the days pass quickly. I always work best when I am in pain—unless it is too severe—for it helps me to forget it."

A school teacher, whose work compelled her to leave her bedridden mother alone for the greater part of five days in each week, had made for her what she called a pair of "sick-room tongs." These were a jointed affair that when unfolded to the full would reach out as far as six feet from the bed. The ends were padded, and by means of them the invalid could reach out a long way for anything she needed. Another sufferer had a brother who arranged for him a reading-board—a simple affair of a board and a couple of elastic bands to keep down the pages as he read, which was slung by a pulley from the ceiling. By means of a pulley also, exercising machines for invalids can be made. A rope is slung over the pulley with a weight adapted to the invalid's strength at one side, and the handle of a rug-strap at the other. This affords a convenient arm exercise. For leg exercises a second pulley must be screwed into the floor, and the rope passed through that also, before it is given into the patient's hands. At a convenient place put a padded loop for the foot to rest in, and as the rope is pulled towards the patient the leg will be raised. A clever nurse managed to cheer the monotony of a long convalescence by arranging the mirror in the patient's room so that it reflected what passed in the street. No doubt many other nurses have schemes and contrivances by which they lessen the pain and tedium of their patients' lives. It would be a kind action to narrate them for the benefit of others.

